# Patterns of Age-Associated Degeneration Differ in Shoulder Muscles

**DOI:** 10.3389/fnagi.2015.00236

**Published:** 2015-12-22

**Authors:** Yotam Raz, Jan F. Henseler, Arjen Kolk, Muhammad Riaz, Peer van der Zwaal, Jochem Nagels, Rob G. H. H. Nelissen, Vered Raz

**Affiliations:** ^1^Department of Orthopaedics, Leiden University Medical CenterLeiden, Netherlands; ^2^Department of Molecular Epidemiology, Leiden University Medical CenterLeiden, Netherlands; ^3^Department of Human Genetics, Leiden University Medical CenterLeiden, Netherlands; ^4^Department of Orthopaedic Surgery, Medical Center HaaglandenHague, Netherlands

**Keywords:** muscle atrophy, aging, shoulder, rotator cuff tear, fatty infiltration, MRA, fibrosis, satellite cells

## Abstract

Shoulder complaints are common in the elderly and hamper daily functioning. These complaints are often caused by tears in the muscle-tendon units of the rotator cuff (RC). The four RC muscles stabilize the shoulder joint. While some RC muscles are frequently torn in shoulder complaints others remain intact. The pathological changes in RC muscles are poorly understood. We investigated changes in RC muscle pathology combining radiological and histological procedures. We measured cross sectional area (CSA) and fatty infiltration from Magnetic Resonance Imaging with Arthrography (MRA) in subjects without (*N* = 294) and with (*N* = 109) RC-tears. Normalized muscle CSA of the four RC muscles and the deltoid shoulder muscle were compared and age-associated patterns of muscle atrophy and fatty infiltration were constructed. We identified two distinct age-associated patterns: in the supraspinatus and subscapularis RC muscles CSAs continuously declined throughout adulthood, whereas in the infraspinatus and deltoid reduced CSA was prominent from midlife onwards. In the teres minor, CSA was unchanged with age. Most importantly, age-associated patterns were highly similar between subjects without RC tear and those with RC-tears. This suggests that extensive RC muscle atrophy during aging could contribute to RC pathology. We compared muscle pathology between torn infraspinatus and non-torn teres minor and the deltoid in two patients with a massive RC-tear. In the torn infraspinatus we found pronounced fatty droplets, an increase in extracellular collagen-1, a loss of myosin heavy chain-1 expression in myofibers and an increase in Pax7-positive cells. However, the adjacent intact teres minor and deltoid exhibited healthy muscle features. This suggests that satellite cells and the extracellular matrix may contribute to extensive muscle fibrosis in torn RC. We suggest that torn RC muscles display hallmarks of muscle aging whereas the teres minor could represent an aging-resilient muscle.

## Introduction

Musculoskeletal disorders are highly prevalent in the elderly, leading to substantial hindering of functional mobility and daily functioning. Over half of the individuals above the age of 70 develop chronic shoulder diseases (Picavet and Schouten, 2003). Despite the high impact of RC pathology on daily functioning in the elderly, the effect of aging on the shoulder muscles is poorly understood (Hermans et al., 2013). In previous studies a strong correlation was found between the presence of a RC-tear and age, suggesting that RC muscles are under continuous age-associated stress (Feng et al., 2003; Fehringer et al., 2008; Yamamoto et al., 2010). However, how muscle degeneration in the shoulder changes during aging in the intact RC, as well as in RC-tears remains unclear. Ninety percent of these shoulder complaints are either diagnosed as subacromial pain syndrome (SAPS) or as tears of the stabilizing rotator cuff (RC; Steinfeld et al., 1999; Koester et al., 2005). Although past research on RC-tear mainly focused on the tendons, recently muscle degeneration was also considered to play a causative role (Laron et al., 2012). However, pathophysiology of the RC muscles in tear conditions are poorly understood.

Muscle atrophy, defined by the loss of muscle mass, is associated with loss of muscle strength and increase in fatty infiltration and inflammation (Evans, 2010). Muscle atrophy is highly prominent in the elderly and can distinguish between healthy and frail individuals (Taekema et al., 2012). Muscle atrophy in RC-tears is considered as a clinical determinant for surgical success in RC-tear repair (Tashjian et al., 2010; Mall et al., 2014) and long-term functionality after surgery (Shen et al., 2008). Recently, atrophy of the supraspinatus (SSp) RC muscle has been suggested to have a prominent role in RC-tearing (Barry et al., 2013). The SSp is the foremost affected RC muscle, and so far is the major focus of most studies (Nakagaki et al., 1996; Ashry et al., 2007; Barry et al., 2013). Atrophy of the infraspinatus (ISp) RC muscle was also suggested to contribute in RC diseases (Henseler et al., 2015). As the interplay between all four RC muscles coordinates shoulder movements and stability, it is crucial to consider all four RC muscles to understand the pathogenesis of RC-tears. A description of muscle atrophy and fatty infiltration in the RC could be constructed from non-invasive radiological imaging (Shen et al., 2008; Mall et al., 2014). How muscle pathology changes with age in all RC muscles was not reported. In aging muscles, changes in muscle mass and muscle strength are accompanied by histological changes. Histological changes in torn muscles, albeit frequent in the aging population, are not well-studied. Histological features of aging muscles include extra-cellular matrix (ECM) thickening and fatty infiltration (Brack et al., 2007; Zoico et al., 2010). Whether those pathological marks are also exhibited in torn RC muscles is not fully understood.

The objective of this study was to assess muscle degeneration in both patients with intact and torn RC muscles. Muscle atrophy and fatty infiltration, obtained from Magnetic Resonance imaging with Arthrography (MRA) were used as measures for muscle pathology. We compared patterns of muscle atrophy between subjects without a RC-tear and subjects with a RC-tear for all four RC muscles: SSp, subscapularis (SSc), ISp, and teres minor (Tmi); and the adjacent deltoid (Del). Furthermore, marks of muscle aging were investigated in histological staining comparing torn ISp to non-torn Tmi and Del. Our study suggests that aging-associated histopathological changes differ in skeletal muscles and suggests the Tmi as an aging-resilient muscle.

## Materials and methods

### Study design and participants

A retrospective cross-sectional study was performed on a consecutive series of shoulder MRAs at the orthopedics outpatient clinics in the Medical Center Haaglanden hospitals in the Netherlands between January 1, 2012 and February 13, 2013 (*N* = 442). All patients with atraumatic and chronic shoulder complaints or shoulder instability are routinely evaluated with MRA. Ethical approval was obtained from the Medical Ethics Committee of the Landsteiner Institute, Medical Center Haaglanden for the radiologic evaluations. Since the radiologic evaluations pertain to a retrospective study, the Medical Ethics Committee waived the need for informed consent from the participants included in this study. Four hundred and forty-two shoulder MRAs were identified. Exclusion was based on poor image quality (*N* = 21), presence of a tumor (*N* = 5), isolated biceps tears (*N* = 4), subscapularis tears (*N* = 3), and fractures (*N* = 6). Subjects were grouped according to the absence (*N* = 294) or presence of a RC-tear (*N* = 109) on shoulder MRA. In total, 403 MRAs are included in this study. The RC-tear group included 40 partial SSp tears (53.5 ± 9.5 years old), 57 full thickness SSp tears (54.7 ± 11.7 years old), five full-thickness SSp tears with partial detachment of the ISp tears (63.2 ± 9.6 years old) and seven full-thickness SSp and ISp tears (61.0 ± 9.1 years old). Excluded from the analyses were: 12 images with motion artifacts of the SSc and 29 images with an incomplete field of view of the Del muscle.

Muscle biopsies were collected from two patients with a massive RC-tear of the SSp and the ISp. During tendon transfer surgeries (Henseler et al., 2013, 2014) muscle biopsies of the ISp, Tmi, and Del were obtained. Radiological characteristics of these two patients are detailed in **Table 4**. Medical Ethical approval was obtained from the Medical Ethical Committee of the Leiden University Medical Center for the collection and analyses of the biopsies and informed consent was obtained from the patients involved.

### MRA imaging procedure

Fifteen minutes before MRA, contrast fluid was injected under fluoroscopic guidance into the glenohumeral joint from posterior. All MRAs were performed on Avanto or Symphony MRI units (Siemens AG, Erlangen, Germany) using a dedicated shoulder coil and turbo spin-echo sequences.

Analyses of the images were performed on a PACS Workstation with Sectra IDS5 (Sectra Medical Systems AB, Linköping, Sweden) as monitor readings. As multiple planes and sequences were obtained following the institutional standard shoulder MRA protocol, the T1-weighted transversal and sagittal plane (TR/TE 500-600/11-15, matrix 256; slice thickness 4 mm, inter-slice gap 1 mm, field of view of 15 cm) were systematically evaluated.

**Figure 1:**
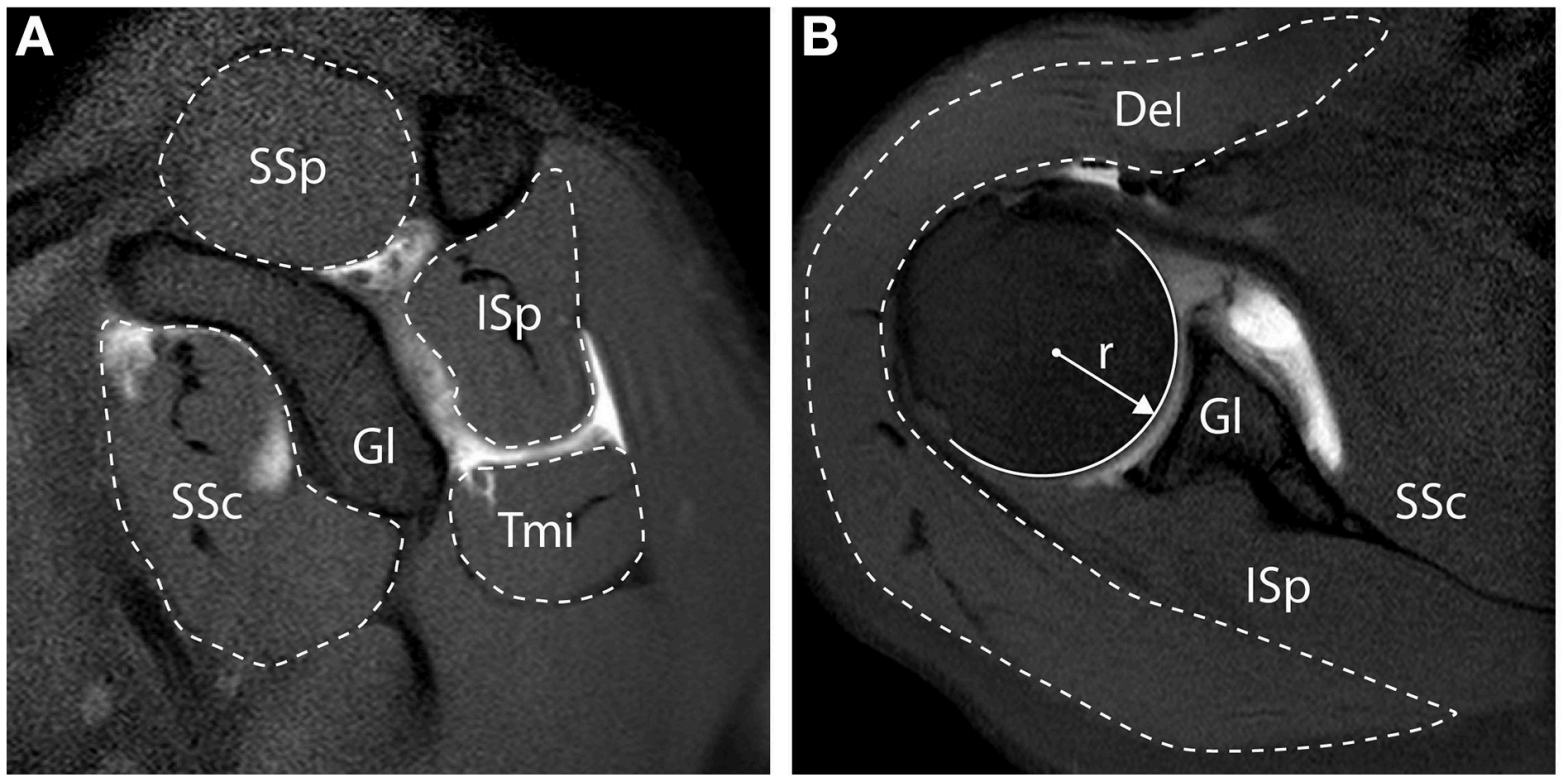
**Measurements of muscle cross-sectional surface area from MR Arthography. (A)** Sagittal view. Cross-sectional surface areas (CSA) of the supraspinatus (SSp), infraspinatus (ISp), teres minor (Tmi), and subscapularis (SSc), relative to the glenoid (Gl) are depicted. **(B)** Transversal view. CSA of the deltoid (Del) is depicted. All CSAs were normalized to the surface of the humeral head, calculated based on the radius of the humeral head (*r*).

Muscle cross sectional area (CSA) quantification, was described previously (Henseler et al., 2015), and examples are shown in Figure 1. In brief, the radius (*r*) of the humeral head at the widest point was measured from its widest point using a circle fit in the transversal plane, and is reported in millimeters (mm). The RC muscles (i.e., SSp, SSc, ISp, Tmi) CSA were measured from the sagittal slice with the anatomical glenoid neck and base of the coracoid present, as illustrated in Figure 1A, and reported in mm^2^. The Del was measured from the transversal slice with the humeral head at its widest point, as illustrated in Figure 1B, and reported in mm^2^. Muscle CSA was normalized to the humeral head surface (in mm^2^), in order to correct for inter-individual anthropometric differences.

The presence of fatty infiltration of the RC was evaluated by examining the presence of intramuscular fatty infiltration in the SSp, SSc, ISp, and Tmi muscles on the sagittal T1-weighted images, and was scored according to the Goutallier score (1, no fatty infiltration; 2, < 50% fatty infiltration; 3, about 50% fatty infiltration; 4, more than 50% fatty infiltration; Goutallier et al., 1994).

### Muscle biopsies

During operation biopsies were collected from the Del, ISp, and Tmi muscles and were immediately frozen in liquid nitrogen and stored at −80°C. Cryosections (16 μm thick) were made with the CM3050-S cryostat (Leica, Solms, Germany) on dry ice and pasted on Superfrost plus glass slides (Menzel-Gläser, Braunschweig, Germany). Sections were stored at −20°C prior to staining. Histological procedures included: (1) Gomori-Trichrome staining (Gomori, 1950). (2) Immunostaining for collagen with goat-collagen-type I (1:200, SouthernBiotech, Birmingham, Alabama, USA), and for satellite cells with mouse-Pax7 antibodies (1:75, Developmental Studies Hybridoma Bank (DSHB), Iowa City, Iowa, USA). Primary antibodies were detected with secondary anti-goat-Alexa-594 or anti-mouse-Alexa-488 (1:1000, Molecular Probes, Invitrogen, Waltham, Massachusetts, USA), respectively. (3) Nile red (1 μM, Sigma-Aldrich, Saint Louis, Missouri, USA) detecting fatty droplets. (4) Immunostaining for myosin heavy chain (MyHC) isotypes was carried out in two sequential steps: first tissues were incubated with monoclonal antibodies detecting MyHC-2x (1:5, hybridoma 6H1, DSHB) and laminin (1:1000, Abcam, Cambridge, UK), followed by secondary anti-mouse-Alexa-546 or anti-rabbit-Alexa-647 (1:1000, Molecular Probes), respectively. Subsequently tissues were incubated with a mixture of monoclonal antibodies to MyHC-1 and MyHC-2a [hybridoma BA-D5 and SC-71, respectively (DSHB)], which are conjugated to Alexa-350 and Alexa-594, respectively, washed and mounted. Conjugation was carried out as previously described (Gregorevic et al., 2008). All sections were treated on the same day with the same antibody mix. Slides were mounted with Aqua Polymount (Polyscience, Niles, Illinois, USA). Nuclei were counterstained with DAPI. Images were generated with either the DM5500 (fluorescent) or DMLB (light) microscopes (Leica, Wetzlar, Germany) using LAS AF software V2.3.6, and for the MyHC immunostaining with the Array scan VTI HCA (Thermofisher, Waltham, Massachusetts, USA). For quantification of Pax7 positive cells, imaging was carried out with a 40X objective.

### Statistical analyses

Differences in characteristics between subjects without or with RC-tears were evaluated with independent *t*-tests and χ^2^-tests. Age-association of CSA was carried out on standardized scores of the normalized CSA. Standardization was performed on the group without and with RC-tears separately. Correlations between standardized scores of the normalized CSA and fatty infiltration (Goutallier score) were evaluated with Pearson correlation tests. Correlations were performed within and in between muscles. Age distribution of subjects with and without RC-tear and in RC-tear shows both a normal distribution and therefore a simple linear regression model corrected for gender was applied to assess age-associated changes. The beta (β) and Pearson correlation coefficient (*R*) were calculated. Visualization of age-related trends in standardized CSA and fatty infiltration is provided in four age groups for subjects without tears, and similar age groups in RC-tear. Statistical significance was considered with a *p* < 0.05 (two-sided). Statistical analyses were performed with SPSS Statistics (IBM Inc., Armonk, New York, USA).

## Results

### Subject characteristics

Muscle CSA and fatty infiltration were measured in five shoulder muscles from 403 individuals. Subject characteristics were stratified for diagnosis (without or with RC-tear), as the mean age in RC-tear was significantly higher compared to those without RC-tear (Table 1). CSAs of the SSp, ISp, and SSc muscles were significantly lower, and in all five muscles fatty infiltration was significantly higher in the RC-tear group compared with the group without RC-tear.

**Table 1:** **Characteristics of subjects**.

	**Without RC-tear** **RC-tear (*N* = 294)**	**RC-tear** **(*N* = 109)**	***p*-values**
**DEMOGRAPHIC DATA**			
*N*	294	109	
Age (years)	42.1 (14.3)	55.1 (10.8)	<0.001
Female, *N* (%)	115 (39.1)	40 (36.7)	0.73
**RADIOGRAPHIC DATA**			
Surface head of humerus (mm^2^)	1954 (383)	1940 (366)	0.74
**SSp MUSCLE**			
Normalized CSA	0.81 (0.21)	0.60 (0.26)	<0.001
Without fatty infiltration, *N* (%)	232 (78.9)	46 (42.2)	^*^<0.001
With fatty infiltration, *N* (%)	62 (21.1)	63 (57.8)	
**SSc MUSCLE**			
Normalized CSA	1.62 (0.76)	1.43 (0.64)	0.01
Without fatty infiltration, *N* (%)	208 (73.2)	41 (37.6)	^*^<0.001
With fatty infiltration, *N* (%)	76 (26.8)	68 (62.4)	
**ISp MUSCLE**			
Normalized CSA	1.17 (0.32)	0.99 (0.35)	<0.001
Without fatty infiltration, *N* (%)	249 (84.6)	61 (56.0)	^*^<0.001
With fatty infiltration, *N* (%)	45 (15.2)	48 (44.0)	
**Tmi MUSCLE**			
Normalized CSA	0.71 (0.21)	0.71 (0.25)	0.97
Without fatty infiltration, *N* (%)	279 (94.9)	91 (83.5)	^*^0.001
With fatty infiltration, *N* (%)	15 (5.1)	18 (16.5)	
**Del MUSCLE**			
Normalized CSA	6.17 (1.68)	6.24 (1.61)	0.70

Means (SD) are provided unless otherwise stated. Muscle cross-sectional area (CSA) is normalized to the humeral head surface. Fatty infiltration was assessed according to the Goutallier classification score, shown are the number and (%) of patients without (Goutallier 1) or with fatty infiltration (Goutallier 2,3,4). Supraspinatus (SSp), Subscapularis (SSc), Infraspinatus (ISp), Teres minor (Tmi), Deltoid (Del).

Nominal variables between subjects without RC-tear and RC-tear patients were compared with t-tests.

* χ^2^-tests were used to compare fatty infiltration across the Goutallier scores between subjects without RC-tear and RC-tear patients.

### Correlation between muscle atrophy and fatty infiltration

We assessed the correlation between a decrease in muscle CSA and an increase in fatty infiltration as a robust measure for muscle degeneration. Within subjects without RC-tear a significant correlation was found only for the SSp and in the SSc muscles (Table 2A). However, in the RC-tear group, significant correlations were found for all four RC muscles (Table 2B). A decrease in CSA of the SSp correlated with increase in fatty infiltration in the other three RC muscles both in the group without RC-tear and in RC-tears (Tables 2A,B). Additionally in the RC-tear group, a decrease in the CSA of the ISp correlated with an increase in fatty infiltration in the other three RC muscles (Table 2B).

**Table 2:** **Correlations of muscle cross sectional area and fatty infiltration in the four RC muscles**.

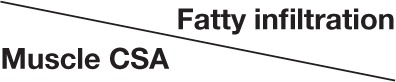	***SSp***		***SSc***		***ISp***		***Tmi***	
	**Pearson correlation**	***p*-values**	**Pearson correlation**	***p*-values**	**Pearson correlation**	***p*-values**	**Pearson correlation**	***p*-values**
**(A) WITHOUT RC-TEAR (***N* = 294**)**								
***SSp***	−**0.33**	<**0.001**	−**0.27**	<**0.001**	−**0.22**	<**0.001**	−**0.20**	**0.001**
***SSc***	−0.08	0.17	−**0.27**	<**0.001**	−0.11	0.07	−0.01	0.92
***ISp***	−0.06	0.34	−0.08	0.17	−0.09	0.12	−0.04	0.53
***Tmi***	−0.05	0.38	−0.02	0.72	−0.06	0.32	−0.11	0.06
**(B) RC-TEAR (***N* = 109**)**								
***SSp***	−**0.66**	<**0.001**	−**0.42**	<**0.001**	−**0.50**	<**0.001**	−**0.23**	**0.02**
***SSc***	−0.18	0.06	−**0.42**	<**0.001**	−**0.23**	**0.02**	−0.18	0.07
***ISp***	−**0.62**	<**0.001**	−**0.34**	<**0.001**	−**0.67**	<**0.001**	−**0.35**	<**0.001**
***Tmi***	0.01	0.95	−0.13	0.19	0.04	0.65	−**0.29**	**0.003**

*Muscle degeneration is assessed by the Pearson correlation between standardized-scores of normalized muscle cross sectional area (CSA) and Goutallier scores of fatty infiltration for each muscle. Panel A shows analyses in subjects without RC-tear; Panel B shows analyses in RC-tear. SSp, Supraspinatus; SSc, Subscapularis; ISp, Infraspinatus, Tmi, Teres minor. Pearson correlations and p-values are provided. Statistically significant correlations are depicted in bold. Correlations between muscle CSA and fatty infiltration within the same muscle are depicted in red*.

### Age-association of muscle atrophy and fatty infiltration

**Figure 2:**
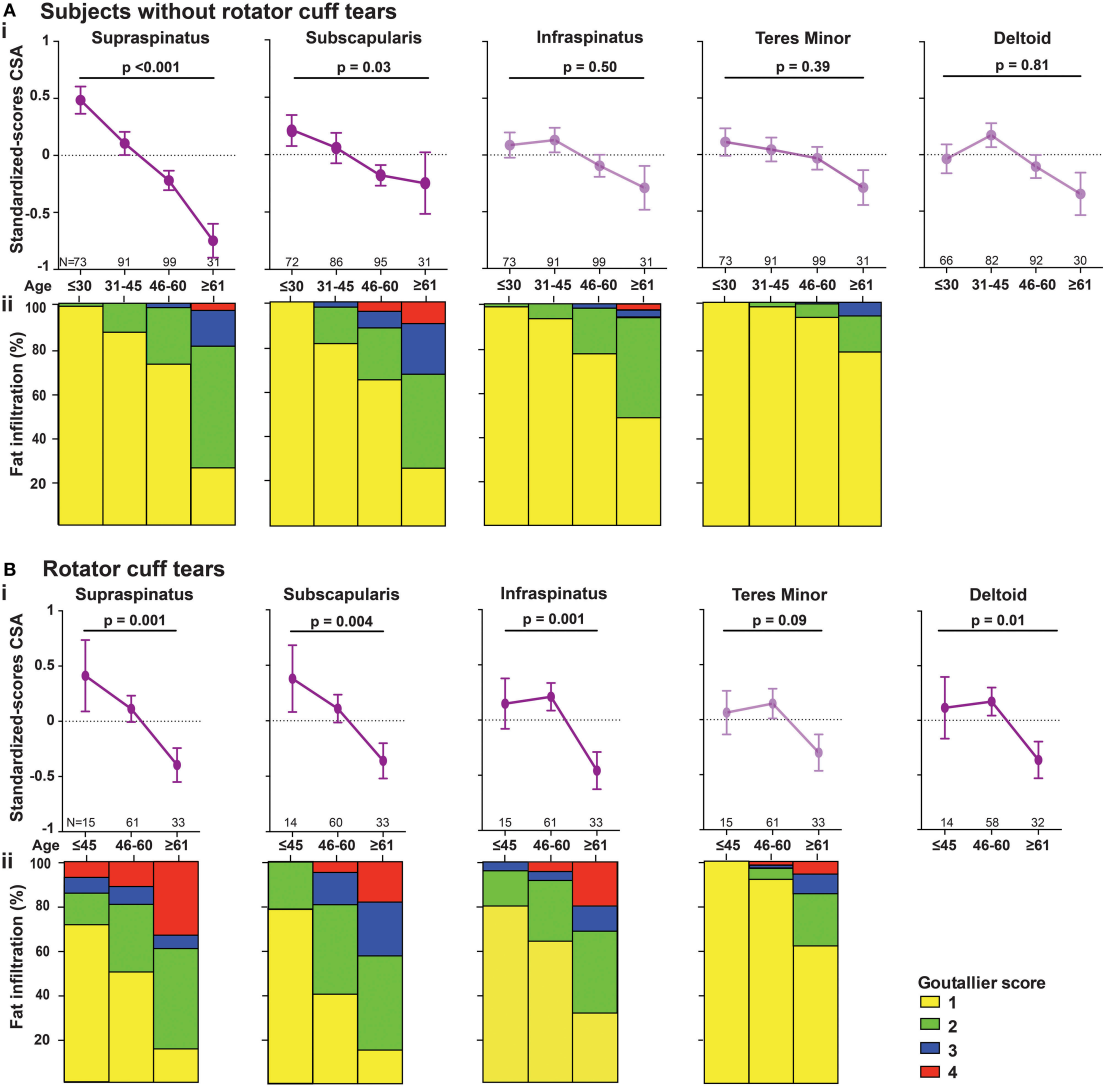
**Age-associated changes in muscle cross sectional area and fatty infiltration in shoulder muscles**. Age-associated analyses were performed in five shoulder muscles (supraspinatus, subscapularis, infraspinatus, teres minor, and deltoid) in subjects without RC-tear **(A)** and in RC-tear **(B)**. (**i**) Age-associated trends of standardized muscle cross-sectional surface areas. *p*-values for age-association were calculated using linear regression and are adjusted for gender. Significant trends are depicted in dark purple, non-significant trends are depicted in light purple. (**ii**) Age-associated increase in fatty infiltration. Fatty infiltration was evaluated according to the Goutallier score.

Age-associated trends of muscle atrophy and fatty infiltration were assessed using a linear regression model, adjusted for gender. In subjects without RC-tears, age-associated decline of SSp and SSc CSA was significant (Table 3). In the SSp and SSc muscles the CSA decreased constantly between 14 and 85 years (Figure 2Ai). The CSA decline in the SSp was 2.6-fold higher compared to that in the SSc. In contrast, in the ISp, Tmi, and Del a decline in muscle CSA was only found from midlife onwards (Figure 2Ai). Fatty infiltration showed an age-associated increase in all five muscles (Table 3), however it was most prominent in the older age group (61–85 years; Figure 2Aii).

**Table 3:** **Age-associated analyses of muscle cross sectional area and fatty infiltration in five muscles of subjects without RC-tear and in RC-tear**.

**Muscle**	**Radiological parameter**	**Without RC-tear (*****N*** = 294**)**			**RC-tear (*****N*** = 109**)**		
		**Beta for age**	***R***	***p*-values**	**Beta for age**	***R***	***p*-values**
*SSp*	Muscle CSA	−**0.021 (0.004)**	**0.38**	<**0.001**	−**0.030 (0.008)**	**0.34**	**0.001**
	Fatty infiltration	**0.018 (0.002)**	**0.51**	<**0.001**	**0.034 (0.009)**	**0.35**	<**0.001**
*SSc*	Muscle CSA	−**0.008 (0.004)**	**0.17**	**0.04**	−**0.026 (0.009)**	**0.38**	**0.003**
	Fatty infiltration	**0.024 (0.003)**	**0.48**	<**0.001**	**0.037 (0.007)**	**0.45**	<**0.001**
*ISp*	Muscle CSA	−0.003 (0.004)	0.25	0.5	−**0.031 (0.008)**	**0.4**	<**0.001**
	Fatty infiltration	**0.014 (0.002)**	**0.46**	<**0.001**	**0.031 (0.008)**	**0.34**	<**0.001**
*Tmi*	Muscle CSA	−0.003 (0.004)	0.18	0.39	−0.015 (0.009)	0.19	0.09
	Fatty infiltration	**0.006 (0.001)**	**0.34**	<**0.001**	**0.018 (0.006)**	**0.32**	**0.001**
*Del*	Muscle CSA	−0.001 (0.122)	0.27	0.81	−**0.023 (0.008)**	**0.43**	**0.007**

*Linear regression analysis was performed to identify age-dependent changes in standardized-scores of normalized muscle cross sectional area (CSA) or in fatty infiltration. The models are adjusted for gender. SSp, Supraspinatus; SSc, Subscapularis; ISp, Infraspinatus; Tmi, Teres minor, Del, Deltoid. Beta (± standard errors) and Pearson correlation coefficient (R) are provided. Statistically significant models are depicted in bold*.

In the RC-tear group, an age-associated decline in muscle CSA was found in the SSp, SSc, ISp, and Del muscles, whereas the Tmi CSA was unaffected (Table 3). In this RC-tear group the SSp muscle was consistently torn in all individuals. However, the decline in the muscle CSA was comparable between the SSp, SSc, and ISp (Table 3). Same as in the non-tear group, also in the RC-tear group CSA declined continuously throughout adulthood, whereas in the ISp and Del it started after midlife (Figure 2i). Moreover, in the RC-tear group an age-associated increase in fatty was found in all five muscles (Table 3, Figure 2ii). Overall, a decline in muscle CSA and an increase in fatty infiltration were both more pronounced in the RC-tear group as compared with the group without RC tear. However, the age-associated patterns were similar between the two groups: a continuous decline in muscle CSA was found for SSp and SSc, but in the ISp and Del the decline started only from midlife onwards (Table 3, Figure 2).

### Histological analyses for muscle degeneration

**Figure 3:**
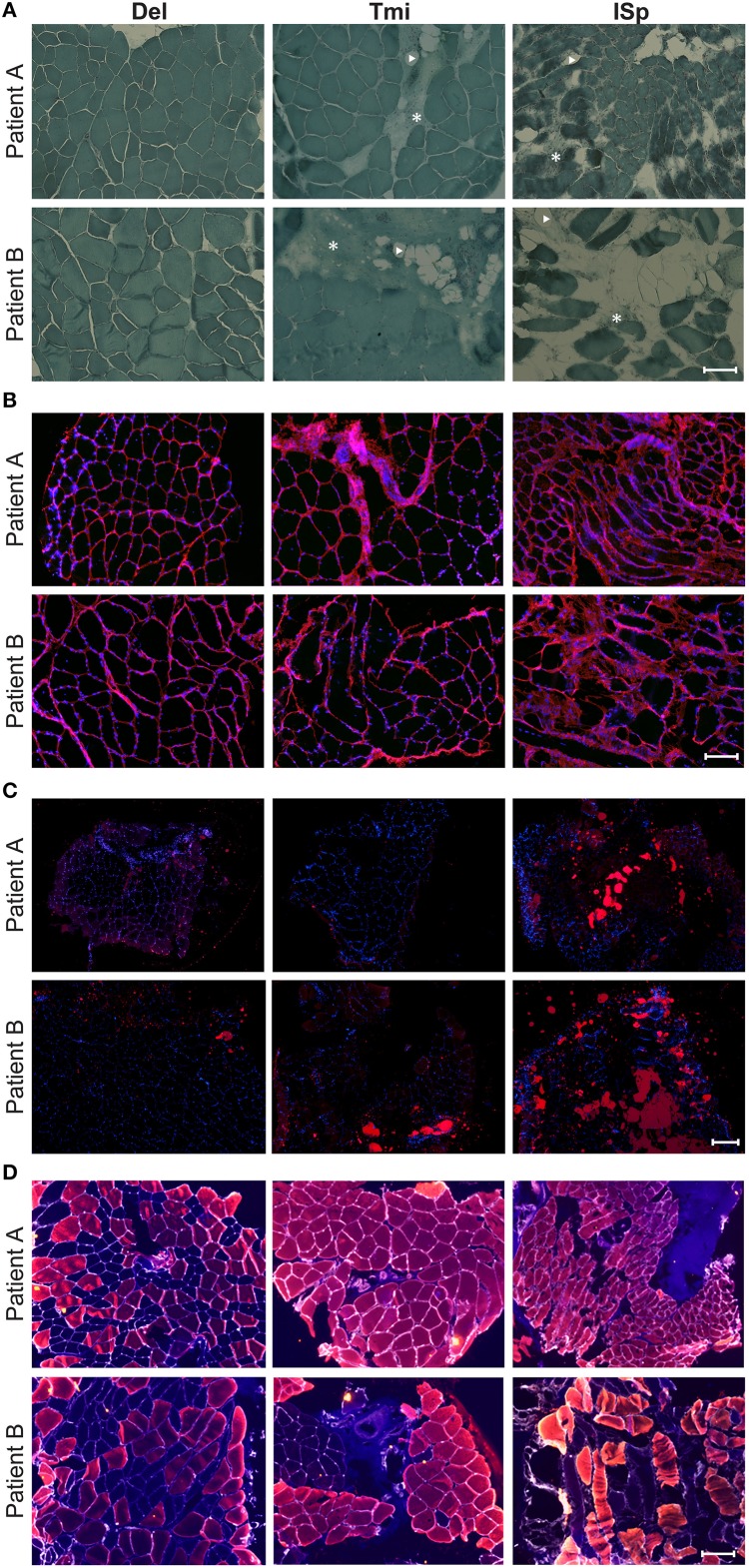
**Histological markers of muscle degeneration in torn and non-torn shoulder muscles**. Representative images of histological analyses of biopsies of torn infraspinatus (ISp), non-torn RC muscle teres minor (Tmi), and deltoid (Del) in two patients. **(A)** Gomori-Trichrome staining shows myofibers in blue-green, nuclei in purple. Fat cells are negatively stained (arrow heads) and fibrotic areas are stained light blue-green (asterisks). Scale bar represents 100 μm. **(B)** Collagen immunostaining (red), nuclei are counterstained with DAPI (blue). Scale bar represents 100 μm. **(C)** Fatty droplet (red) staining, nuclei are counterstained with DAPI (blue). Scale bar represents 200 μm. **(D)** Immunostaining for myosin heavy chain (MyHC) isotypes: MyHC-1 (blue), MyHC-2a (red), MyHC-2x (yellow), and the basal lamina (gray). Scale bar represents 100 μm.

To explore whether the radiological features in RC-tear conditions are accompanied by aging-associated tissue degeneration, we performed histological analyses of muscle biopsies with known aging histopathological marks. Muscle biopsies were obtained from two subjects at comparable age. Both patients had a massive RC tear involving the SSp and ISp. Their radiological characteristics were more severe than the average of the entire RC tear group (Table 4). CSA of SSp and ISp from Patient A were more than 1.5 standard deviations (SD) smaller than the mean of the RC-tear group of the radiological study (Table 4). However, CSA of these muscles from Patient B were within 1 *SD* of the mean of the RC-tear group (Table 4). Overall patient A had more severe muscle atrophy in all five muscles compared to patient B. Histological staining of the torn ISp from both patients showed severe disruption of myofiber orientation, accompanied with fibrosis and fat cells (Figure 3A). In the non-torn Tmi from both patients, fibrosis and fat cells were less prominent compared with the ISp (Figure 3A). In the Del muscle histology was not pathological (Figure 3A). We confirmed extensive fibrosis and thickening of the ECM in the ISp using collagen-1 immunostaining in both patients (Figure 3B). However, ECM thickening was limited in the Tmi and Del from both patients (Figure 3B). This suggests ECM thickening is among the pathological hallmarks of torn RC muscles.

**Table 4:** **Radiological characteristics of subjects for histology**.

	**Patient A (64 years)**	**Patient B (62 years)**
**SSp MUSCLE**		
Normalized CSA (St)	0.21 (–1.52)	0.36 (–0.97)
Goutallier score	2	2
**SSc MUSCLE**		
Normalized CSA (St)	0.83 (–0.92)	1.01 (–0.66)
Goutallier score	1	1
**ISp MUSCLE**		
Normalized CSA (St)	0.22 (–2.15)	0.74 (–0.71)
Goutallier score	3	1
**Tmi MUSCLE**		
Normalized CSA (St)	0.58 (–0.52)	0.88 (+0.67)
Goutallier score	1	1
**Del MUSCLE**		
Normalized CSA (St)	5.29 (–0.60)	9.03 (+1.70)

*Muscle cross-sectional area (CSA) is normalized to the humeral head surface. St, corresponding standardized score in the entire RC-tear group. Fatty infiltration was assessed according to the Goutallier classification score*.

We validated fatty infiltration using Nile-red staining. This confirmed fatty infiltration in the ISp from both patients, but only limited fatty droplet staining was found in Tmi or Del muscles (Figure 3C).

To determine contractile features of those muscles we employed immunostaining for three MyHC isotypes. Laminin staining was used to identify myofiber contour. Laminin staining revealed disruption in myofiber orientation in the torn ISp (Figure 3D). Additionally, we found that while both MyHC-1 and -2a, were expressed in Del muscles, in the Tmi and the torn ISp the expression of MyHC-1 was dramatically reduced. Furthermore, MyHC-2x was co-expressed with MyHC-2a in the torn ISp of patient B (Figure 3D).

**Figure 4:**
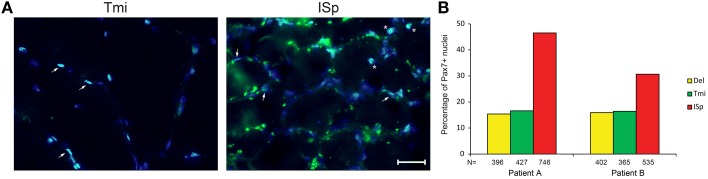
**Satellite cells in torn and non-torn shoulder muscles. (A)** Representative images of Pax7 immunostaining (green) in non-torn RC muscle teres minor (Tmi) and torn infraspinatus (ISp). Nuclei were counter-stained with DAPI (blue). Pax7-positive nuclei have an overlay of blue and green. Examples of Pax7-positive myonuclei are marked with arrowheads. Examples of Pax7-positive nuclei not in myonuclei are marked with asterisks. Scale bar represents 25 μm. **(B)** Bar charts for the quantification of Pax7-positive nuclei in deltoid, teres minor and infraspinatus sections of two patients. *N* represents the number of total nuclei analyzed for each muscle in 11 fields.

We also explored the regenerative capacity of torn RC muscles. Muscle sections were immunostained with an anti-Pax7 antibody, marking satellite cells. We found a two- to three-fold increase in the fraction of Pax7-positive nuclei in torn ISp compared to Del or Tmi (Figures 4A,B). The fraction of Pax7-positive nuclei between Del and Tmi was similar (Figure 4B). Furthermore, in the Del and Tmi all Pax7 staining overlaid within myonuclei, whereas in the torn ISp Pax7 staining was also found outside myonuclei (Figure 4A).

Overall, histological analyses confirmed that in both patients the torn ISp is severely degenerated compared to the non-torn Tmi and Del muscles. Some histological differences were found between the two patients, especially with MyHC isotypes expression. Overall in both patients histological features of the Tmi RC muscle were comparable to Del muscles rather than to ISp RC-muscle.

## Discussion

Aging-associated changes in skeletal muscles are prominent in part because it is the most abundant in the human body. There are over 400 skeletal muscles in the human body and how their pathology changes during aging is largely unknown. So far most studies are carried out on vastus lateralis using cross-sectional studies. In vastus lateralis an aging-associated decline in muscle strength and muscle mass starts at the sixth decade (Williams et al., 2002; Faulkner et al., 2007). Here we found that muscle atrophy in ISp and Del muscles starts only after the age of 45, while a continuous decline throughout adulthood was found in the SSp and SSc. This indicates that mechanisms leading to muscle atrophy could be similar between vastus lateralis, ISp and Del muscles, but are likely to differ from those regulating muscle atrophy in SSp and SSc. In contrast, in the Tmi, muscle atrophy did not change significantly with age, suggesting this muscle is less susceptible for age-associated changes. Since the Tmi is unaffected in RC-tears (Melis et al., 2011), it is poorly studied. We suggest that the Tmi could represent an aging-resilient muscle. The histology of Tmi muscle from two patients with a massive RC-tear was also showed a healthy histology, similar to that of Del muscles within the same patient. In contrast, the torn ISp exhibited aging and degenerated muscle pathology. The Tmi muscle could be used to identify potential molecular regulators that protect skeletal muscles from damage during aging.

Torn muscles are often characterized by atrophy and fatty infiltration (Goutallier et al., 1994; Barry et al., 2013). We confirmed this increase in fatty infiltration in torn muscles using fatty droplets staining. Additionally, we found ECM thickening in torn muscles, indicating fibrosis. These features are also common in aging muscles (Brack et al., 2007; Zoico et al., 2010), suggesting that torn RC muscles and aging muscles share pathological mechanisms. However, this should be confirmed by additional studies. Changes in the contractile function is marked by the expression of MyHC isotypes, which can be changed in aging and in disease (Ciciliot et al., 2013). Fiber type transitions can vary between skeletal muscles, presumably due to different functions (Ciciliot et al., 2013). A transition from fast (type 1) to slow (type 2) myofibers is often found in muscular dystrophies and in metabolic disorders (Ciciliot et al., 2013). In the lower limb, type-2 myofibers decrease in aging vastus lateralis muscles (Verdijk et al., 2007). However, a transition from slow (type-1) to fast (type-2) myofibers is found in muscle disuse conditions, including denervation and loss of tensile strength (Ciciliot et al., 2013). The myofiber transition in torn RC is not well-characterized. In the intact RC MyHC type-1, -2a, and -2x are expressed in all four RC muscles (Lovering and Russ, 2008). Reduced MyHC-1 has been reported in cases with a severely torn SSp (Lundgreen et al., 2013). In agreement with that study, we also found a prominent loss of type-1 MyHC in torn ISp. Moreover, in severe denervation conditions (e.g., spinal cord injury) a myofiber switch from slow to fast fiber type was found (Verdijk et al., 2012) and is also consistent with our findings in torn ISp. Interestingly, the axillary nerve innervates the Tmi, but the SSp and ISp are innervated by the suprascapular nerve. Recent hypotheses suggest a role for denervation in torn RC muscles (Gigliotti et al., 2015). This calls for additional studies on the role of denervation in RC-tear and in muscles preserved from tearing.

In addition, we found that severely degenerated ISp muscles have an increased number of Pax7-positive cells. However, this increase may not represent an increase of satellite cells as some of the Pax7-staining was not within myonuclei. An increase in Pax7-positive cells was also reported in affected muscles from oculopharyngeal muscular dystrophy (OPMD; Gidaro et al., 2013). OPMD is a slow progressive myopathy, which could represent accelerated muscle aging (Raz and Raz, 2014). Satellite cells in chronic and slow progressive conditions were suggested to suppress muscle regeneration, possibly by remaining dormant and by differentiating into fibrogenic cells (Brack et al., 2007; Sciorati et al., 2015). Moreover, an adverse local environment could contribute to decreased regeneration by satellite cells (Meng et al., 2015). Although we analyzed biopsies from only two patients, as comparisons were performed within the same patient our findings likely represent degenerative changes between torn and non-torn RC muscles. Future studies with a larger sample size should investigate degenerative changes in torn RC muscles.

We also found an age association of fatty infiltration in subjects without an RC-tear however this was mostly contributed by the elderly group. Consistent with previous studies (Goutallier et al., 1995; Gerber et al., 2007; Gladstone et al., 2007), our results also show that fatty infiltration in RC-tear is highly prominent. In this cross-sectional study muscle atrophy appears at earlier age compared with fatty infiltration. This suggests that muscle atrophy in the RC develops earlier than fatty infiltration, while fatty infiltration in unaffected muscles is possibly systemic. Although our evaluation of fatty infiltration from MRA is qualitative, it is in agreement with a quantitative radiological study, where similar age-association of fatty infiltration was found in torn SSp (Nozaki et al., 2015).

Although muscle atrophy and fatty infiltration are both increased in aging how they are interrelated is not fully understood. In subjects without RC-tear we found that muscle atrophy correlated with fatty infiltration only in the SSp and SSc muscles. However, in RC-tear due to higher atrophy and fatty infiltration, correlations were found within all four RC muscles. In subjects without RC-tear we found that the SSp atrophy correlated with fatty infiltration in all other three RC muscles. Those correlations between muscle atrophy and fatty infiltration with the other three muscles were expanded to the ISp in RC-tear. This suggests that muscle atrophy in the SSp may affect muscle degeneration in the adjacent RC muscles.

Comparable trends of muscle atrophy were found in subjects without as well as with a RC-tear. Although the trends in muscle atrophy with age are similar between subjects without and with a RC-tear, the atrophy of the RC muscles overall was larger in the RC-tear group. In agreement, age-associated muscle atrophy in the SSp in both groups was reported, but was more pronounced in RC-tears (Barry et al., 2013). This suggests accelerated muscle atrophy in RC-tears. It is unclear whether muscle wasting in the RC is a consequence or cause of RC-tear. Longitudinal studies could further reveal the causality of muscle atrophy in RC-tear.

We conclude that patterns of age-associated degeneration differ in skeletal muscles of the RC. While some RC muscles show continuous changes throughout adulthood, in others changes start only from midlife onwards. Whereas, the majority of RC muscles show age-associated changes, the teres minor did not show significant age-associated changes. In torn RC muscles satellite cells and the ECM are increased compared to the intact teres minor. We propose that torn RC muscles display hallmarks of muscle aging whereas the teres minor could represent an aging-resilient muscle, suggesting a role of muscle pathology in RC tear pathogenesis.

## Author contributions

YR and JH measured and analyzed MRA images and wrote the MS. MRA images were provided by PvdZ. Biopsies were collected by JFH, AK, and JN. Sectioning of biopsies, histological staining and imaging were performed by YR, MR, and VR. RN and VR supervised the project. All authors contributed to the writing and discussions of the results. All authors read and approved the final manuscript.

## Funding

This study is partly funded by the Dutch Arthritis Association (DAA), grant number RF 13-1-303.

### Conflict of interest statement

The authors declare that the research was conducted in the absence of any commercial or financial relationships that could be construed as a potential conflict of interest.
